# Feasibility and diagnostic performance of PETRA-MRA for postoperative follow-up of intracranial aneurysms: a prospective comparative study

**DOI:** 10.3389/fneur.2026.1786151

**Published:** 2026-04-13

**Authors:** Qin Jiang, Xiao Wang, Jinbing Zhao, Jun Hu

**Affiliations:** 1 Department of Radiology, The Affiliated Brain Hospital of Nanjing Medical University, Nanjing, Jiangsu, China; 2 Department of Neurosurgery, The Affiliated Brain Hospital of Nanjing Medical University, Nanjing, Jiangsu, China

**Keywords:** flow diverter, intracranial aneurysm, PETRA-MRA, postoperative follow-up, stent-assisted coiling

## Abstract

**Objective:**

Metal-related susceptibility artifacts have long limited the use of magnetic resonance angiography (MRA) for postoperative surveillance after intracranial aneurysm interventions. Pointwise encoding time reduction with radial acquisition (PETRA) substantially reduces metal-induced artifacts. This study aimed to evaluate the performance of PETRA-MRA in depicting parent artery image quality and aneurysm occlusion status at postoperative day 1 (T0) and 6 months (T1), and to explore its feasibility as a noninvasive alternative to digital subtraction angiography (DSA) for follow-up.

**Methods:**

In this prospective study, 100 patients harboring 100 intracranial aneurysms underwent time-of-flight MRA (TOF-MRA), PETRA-MRA, and DSA at both T0 and T1. Parent artery image quality was compared across time points and treatment modalities stent-assisted coiling (SAC) vs. flow diverter (FD) placement alone. A parsimonious cumulative logit regression model was performed to identify factors associated with PETRA-MRA image quality. Using DSA as the reference standard, the sensitivity, specificity, and accuracy of PETRA-MRA for assessing aneurysm occlusion were calculated.

**Results:**

Across both treatment groups, PETRA-MRA demonstrated significantly improved image quality at T1 compared with T0. At T1, both TOF-MRA and PETRA-MRA yielded higher image quality scores in the FD group than in the SAC group. Regression analysis indicated that none of the pre-specified covariates demonstrated a statistically significant independent association with PETRA-MRA image quality at T1 based on bootstrap-based inference. Complete occlusion rates increased markedly from T0 to T1: from 21.4% to 71.4% in the FD group and from 80.6% to 91.7% in the SAC group. Using DSA as the reference standard, PETRA-MRA demonstrated higher diagnostic accuracy than TOF-MRA at both time points in the SAC group, with accuracies of 94.44% in T0 and 97.67% in T1.

**Conclusion:**

PETRA-MRA demonstrates improved diagnostic performance at mid-term follow-up compared with the early postoperative period. Its marked ability to reduce metal artifacts and enhance visualization of both the parent artery and aneurysm lumen supports its potential as an effective, noninvasive alternative to DSA for long-term surveillance after intracranial aneurysm treatment.

## 1 Introduction

Endovascular treatment of intracranial aneurysms, including stent-assisted coiling (SAC) and flow diverter (FD) placement, has become an essential strategy for preventing aneurysm rupture and rebleeding (1–4). Compared with microsurgical clipping, endovascular therapy yields a higher disability-free survival rate at 1 year (5), and this advantage persists for at least 7 years (6). However, recent studies have reported an early postoperative complication rate of 21.6% and a long-term incomplete occlusion rate of 26.9% following stent-based treatment (7), indicating that some patients may require surveillance extending for more than 10 years (8). Moreover, given the elevated risk of restenosis after intracranial stenting in patients with atherosclerotic disease (9), the long-term vascular risks after SAC also warrant attention. Consequently, postoperative imaging follow-up is crucial for assessing parent artery patency and aneurysm occlusion. Although digital subtraction angiography (DSA) remains the reference standard, its invasive nature, radiation exposure, and need for iodinated contrast limit its feasibility for long-term or repeated follow-up.

Magnetic resonance angiography (MRA)—particularly three-dimensional time-of-flight MRA (TOF-MRA)—is widely used for post-treatment evaluation because it is noninvasive and highly reproducible. Nonetheless, magnetic susceptibility artifacts caused by intracranial stents frequently obscure the parent artery and result in signal dropout, complicating the assessment of in-stent flow and residual aneurysm filling. Pointwise Encoding Time Reduction with Radial Acquisition (PETRA-MRA), an emerging technique, generates non-contrast vascular images by subtracting pre- and post-blood saturation acquisitions (10, 11). Its ultrashort echo time (UTE)-MRA substantially reduces metal artifacts and improves visualization of vascular structures adjacent to stents. Prior research has shown that PETRA-MRA provides superior image quality to TOF-MRA in the follow-up of intracranial arterial stenosis and can accurately assess middle cerebral artery stenosis and in-stent lumen anatomy (12). These findings suggest that PETRA-MRA may serve as a valuable adjunct—or even a noninvasive alternative—to DSA during postoperative surveillance.

However, existing studies on the use of PETRA-MRA after endovascular treatment of intracranial aneurysms remain limited (13, 14), and most have focused primarily on image quality or small retrospective cohorts. Few studies have systematically evaluated its diagnostic performance against the reference standard of DSA, compared its performance across different treatment modalities (FD vs. SAC), or examined imaging findings at different postoperative follow-up stages. Whether PETRA-MRA can reliably evaluate long-term aneurysm occlusion and how closely it approximates DSA findings therefore remain incompletely understood. Importantly, local hemodynamics differ considerably between the early postoperative period and long-term follow-up. As endothelialization progresses and blood flow becomes more stable, the visibility of stented segments on PETRA-MRA may improve over time, suggesting that imaging performance may vary across follow-up stages.

Based on these considerations, the present study systematically evaluated PETRA-MRA, TOF-MRA, and DSA at postoperative day 1 (T0) and 6 months (T1) in patients undergoing endovascular treatment for intracranial aneurysms (15). We compared image quality and diagnostic agreement across treatment types and investigated the potential value of PETRA-MRA as a long-term, noninvasive follow-up strategy.

## 2 Methods

### 2.1 Patients

This prospective cohort study included 110 patients with intracranial aneurysms who underwent endovascular treatment at Nanjing Medical University Affiliated Brain Hospital between December 2023 and October 2025. During the 6-month follow-up period, 10 patients were lost to follow-up (attrition rate 9.1%). Ultimately, 100 patients with a total of 100 aneurysms completed all scheduled examinations and were included in the final analysis. Inclusion criteria were as follows: (1) Patients diagnosed with intracranial aneurysms who underwent endovascular treatment at our institution and were scheduled for DSA follow-up; (2) Age ≥ 18 years; (3) No contraindications to MRA or DSA. Exclusion criteria included: (1) Patients treated with parent artery occlusion or surgical aneurysm clipping; (2) Severe systemic comorbidities (e.g., cardiac, pulmonary, hepatic, or renal failure); (3) Known allergy to iodinated contrast agents; (4) Contraindications to MRI (e.g., incompatible implants, severe claustrophobia); (5) Images with severe artifacts (e.g., from excessive motion) precluding diagnostic evaluation.

The imaging follow-up workflow was predefined according to institutional clinical practice protocols. All patients underwent follow-up examinations at T0 and T1. At each time point, TOF-MRA, PETRA-MRA, and DSA examinations were performed in sequence, with the interval between MRA and DSA limited to ≤24 h. Image quality assessment of the parent artery and diagnostic performance for aneurysm occlusion were defined as the primary evaluation outcomes of the study. Because DSA constituted part of routine clinical assessment, no additional procedural risks were imposed on participants. This study was granted approval by the Ethics Committee of Brain Hospital affiliated with Nanjing Medical University and written informed consent was obtained from all participants prior to enrollment.

### 2.2 Image acquisition

All MRI examinations were performed on a 3.0-T Siemens Prisma scanner using a 64-channel head–neck coil. Patients were positioned supine. Scan parameters were as follows: TOF-MRA: repetition time (TR) = 20 ms, echo time (TE) = 3.3 ms, field of view (FOV) = 220 × 200 mm^2^, matrix = 384 × 288, voxel size = 0.6 × 0.6 × 0.6 mm^3^, slice thickness = 0.60 mm, flip angle (FA) = 20°, number of slab = 6, slices per slab = 36, acquisition time = 4 min 39 s. PETRA-MRA: TR = 3.32 ms, TE = 0.07 ms, FOV = 256 × 256 mm^2^, matrix = 320 × 320, radial views = 30,000, voxel size = 0.8 × 0.8 × 0.8 mm^3^, slice thickness = 0.8 mm, FA = 6°, number of slab = 1, slices per slab = 320. Acquisition time for the non-labeled sequence was 1 min 50 s and for the labeled sequence 3 min 4 s. The final angiographic images were generated by subtracting non-labeled images from labeled images.

### 2.3 DSA acquisition

All DSA examinations were performed on a Philips FD 20 Artis system. Femoral artery access was obtained using a modified Seldinger technique, and a 5F arterial sheath was inserted. Under guidance of a 0.038-inch hydrophilic wire, a 5F single-curve catheter was placed into the target vessel. Bilateral internal carotid, external carotid, and vertebral artery angiography was performed. Iodixanol contrast medium (350 mgI/mL) was injected at 5 mL/s using a power injector, with a total volume of 8 mL per acquisition. Rotational angiography was performed over 200°, with an FOV of 320 × 320 mm^2^, matrix of 1,024 × 1,024, and 133 frames acquired. All patients underwent standard anteroposterior, lateral, working-projection, and three-dimensional (3D) rotational angiography as part of the imaging protocol.

### 2.4 Image analysis

All MRA and DSA images were anonymized and randomly coded. Two neuroradiologists, each with more than 10 years of experience, independently evaluated the images. Assessment parameters included the image quality of the parent artery and the aneurysm occlusion status. Radiologists were permitted to review raw images, maximum-intensity-projection images, and volume-rendered reconstructions. The radiologists who evaluated the MRA images were blinded to the DSA findings and clinical information during image assessment.

To minimize recall bias, PETRA-MRA and TOF-MRA were evaluated at least 2 weeks apart. Two weeks after completing MRA interpretation, the same radiologists evaluated aneurysm occlusion on DSA.

Parent artery image quality was rated on a 5-point scale (16): 1 = no visualization; 2 = poor visualization with severe blurring or artifacts; 3 = acceptable visualization with moderate blurring/artifacts but diagnostic; 4 = good visualization with only mild artifacts; 5 = excellent visualization with clear vessel depiction and no artifacts. If ratings differed, the mean of two scores was used.

Aneurysm occlusion was assessed dichotomously (17) as: Complete occlusion (no visualization of the neck or sac), or Incomplete occlusion (residual filling of the neck and/or sac). Discrepancies were resolved by consensus.

### 2.5 Statistical analysis

Statistical analyses were performed using SPSS version 26.0. Continuous variables were presented as mean ± standard deviation, and categorical variables as frequencies (percentages). Image quality scores for TOF-MRA and PETRA-MRA, which were ordinal variables, were summarized as median (interquartile range). Intragroup and intergroup comparisons (e.g., TOF-MRA vs. PETRA-MRA; T0 vs. T1) were conducted using the Wilcoxon signed-rank test. All multiple pairwise comparisons were adjusted using the Benjamini–Hochberg false discovery rate (FDR) procedure with *q* = 0.05. Inter-observer agreement was evaluated using Cohen’s *κ* coefficient.

To minimize overfitting given the limited sample size, we fitted a parsimonious ordinal logistic regression model (cumulative logit) including a limited set of clinically pre-specified covariates (≤4 predictors). The PETRA-MRA image quality score at T1 was used as the dependent variable. Pre-specified covariates included segment category (ophthalmic, posterior communicating, and other segments), stent type (Enterprise vs. non-Enterprise), maximum aneurysm diameter, and age. Bootstrap resampling (1,000 iterations) was applied to derive robust 95% confidence intervals for model estimates.

Diagnostic performance analyses were conducted using DSA as the reference standard. Sensitivity, specificity, positive predictive value, negative predictive value, and overall accuracy were calculated for TOF-MRA and PETRA-MRA in detecting residual aneurysm filling.

## 3 Results

### 3.1 Patient demographics and baseline characteristics

**Figure 1:**
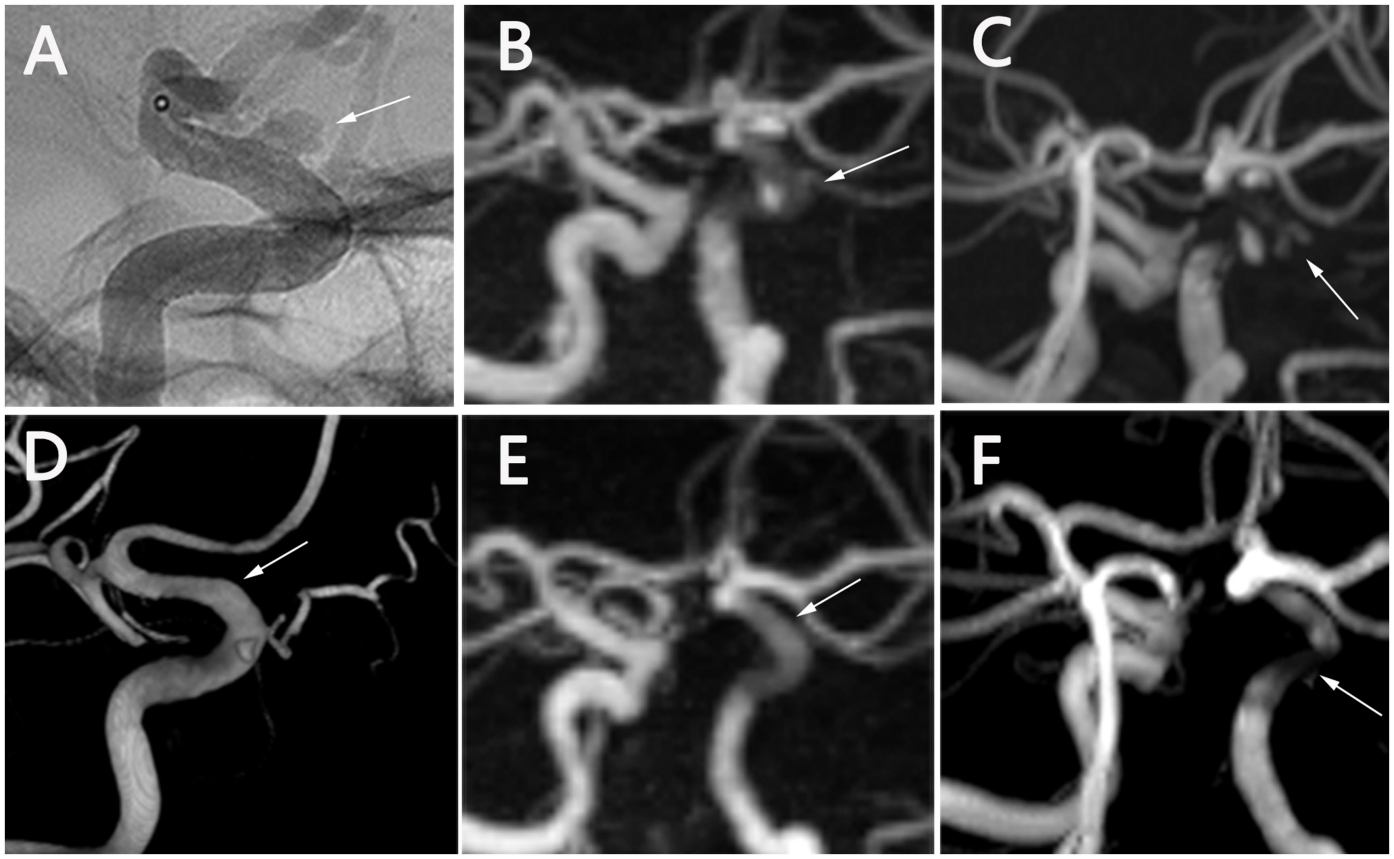
Follow-up images of a case with a FD device for an aneurysm in the paraophthalmic segment of the left internal carotid artery. **(A–C)** DSA shows a clear view of the aneurysm at T0 **(A)**. PETRA-MRA can display the residual aneurysm well **(B)**, whereas TOF-MRA shows significant flow voids affected by artifacts **(C)**. **(D–F)** DSA shows the aneurysm has occluded and the parent artery remains patent at T1 **(D)**. PETRA-MRA clearly indicates complete occlusion, consistent with DSA **(E)**. TOF-MRA still shows noticeable signal interruption **(F)**. T0, 1 day postoperatively; T1, 6 months postoperatively; FD, flow diversion; DSA, digital subtraction angiography; PETRA-MRA, pointwise encoding time reduction with radial acquisition magnetic resonance angiography; TOF-MRA, time-of-flight magnetic resonance angiography.

**Figure 2:**
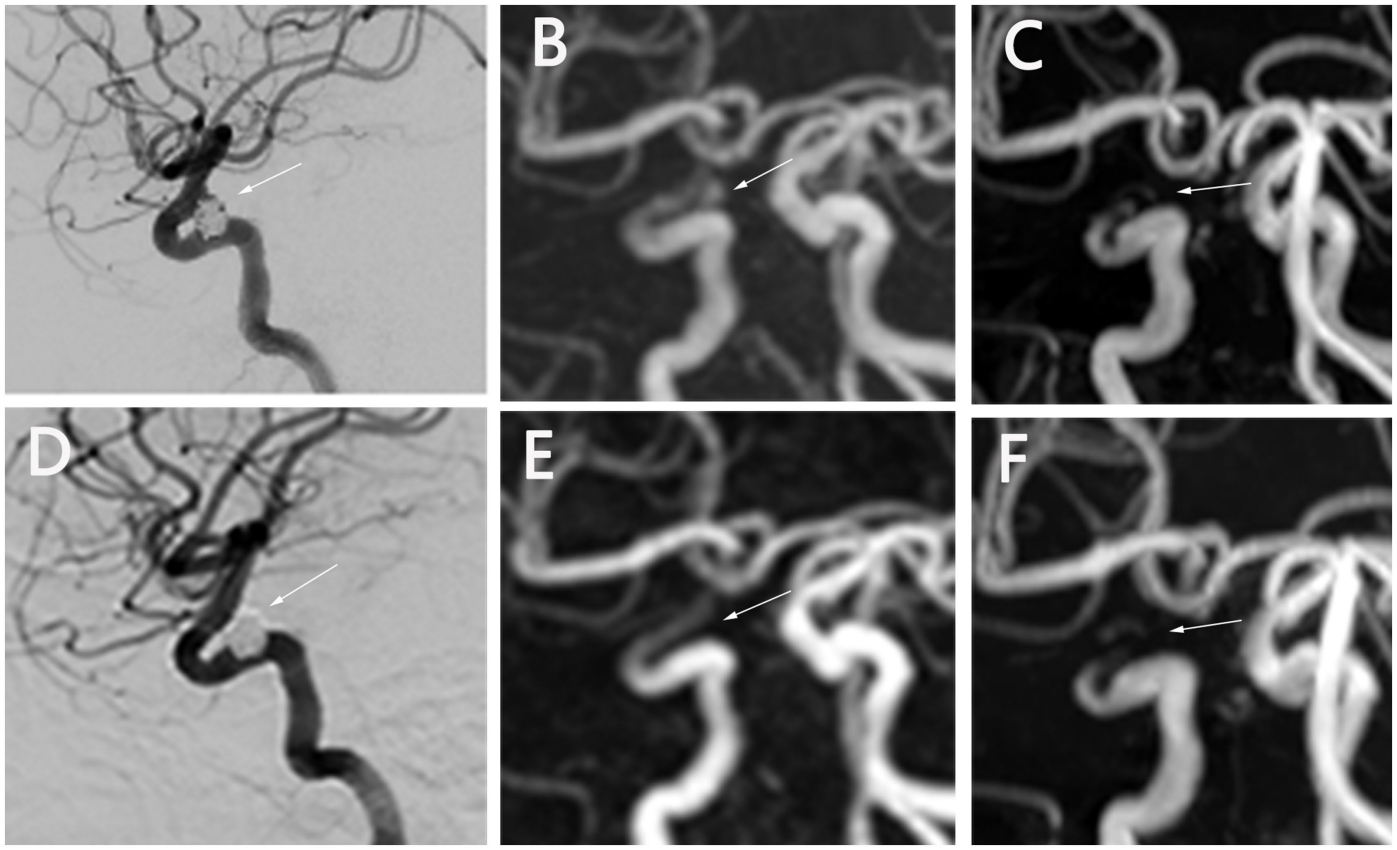
Status of embolization of a right internal carotid artery ophthalmic segment aneurysm after SAC. **(A–C)** DSA shows the aneurysm clearly at T0 **(A)**. PETRA-MRA visualizes blood flow within the aneurysm, consistent with DSA **(B)**. TOF-MRA shows local signal loss **(C)**. **(D–F)** DSA shows patent blood flow, with no aneurysm visible at T1 **(D)**. PETRA-MRA shows the parent artery is patent and the aneurysm is occluded **(E)**. TOF-MRA shows artifacts and vascular discontinuity **(F)**. T0, 1 day postoperatively; T1, 6 months postoperatively; SAC, stent-assisted coiling. DSA, digital subtraction angiography; PETRA-MRA, pointwise encoding time reduction with radial acquisition magnetic resonance angiography; TOF-MRA, time-of-flight magnetic resonance angiography.

A total of 100 patients with 100 intracranial aneurysms were included in the final analysis. Among them, 72 aneurysms (72%) were treated with SAC, and 28 aneurysms (28%) were treated with flow-diverter placement. Typical postoperative images of SAC and those of flow-diverter placement are shown in Figures 1, 2, respectively. Demographic and imaging characteristics are summarized in Table 1.

**Table 1:** Demographic and clinical characteristics of patients with intracranial aneurysms.

Characteristics	Stent-assisted coil embolization group (*n* = 72, 72%)	Standalone flow diverter stent group (*n* = 28, 28%)
Age (years, *x̄*±s)	57.53 ± 10.00	54.14 ± 7.70
Gender, *n* (%)		
Female	46 (63.9)	14 (50.0)
Male	26 (36.1)	14 (50.0)
Aneurysm neck diameter (mm, *x̄*±s)	3.74 ± 1.60	4.81 ± 2.21
Aneurysm size (mm, *x̄*±s)	5.56 ± 3.70	6.79 ± 2.93
Proximal diameter of parent artery (mm, *x̄*±s)	3.26 ± 0.80	3.55 ± 0.98
Distal diameter of parent artery (mm, *x̄*±s)	2.97 ± 0.80	4.04 ± 0.77
Comorbid risk factors, *n* (%)		
Hypertension	32 (44.4)	12 (42.9)
Diabetes mellitus	5 (6.9)	4 (14.3)
Hyperlipidemia	6 (8.3)	2 (7.1)
Coronary heart disease	2 (2.8)	0 (0.0)
Smoking	12 (16.7)	8 (28.5)
Alcohol consumption	7 (9.7)	4 (14.2)
Stent type, *n* (%)		
Laser-engraved stent (Enterprise)	48 (66.7)	—
Laser-engraved stent (Neuroform Atlas)	20 (27.8)	—
Flow diverter stent (Pipeline Flex)	3 (4.2)	22 (78.6)
Flow diverter stent (Lattice)	1 (1.4)	6 (21.4)
Aneurysm location, *n* (%)		
Internal carotid artery ophthalmic segment	36 (50.0)	18 (64.3)
Internal carotid artery posterior communicating segment	14 (19.4)	—
Internal carotid artery cavernous sinus segment	2 (2.8)	—
Middle cerebral artery	8 (11.1)	—
Anterior cerebral artery	4 (5.6)	—
Anterior communicating artery	2 (2.8)	—
Basilar artery	5 (6.9)	—
Posterior cerebral artery	1 (1.4)	—
Vertebral artery V4 segment	—	10 (35.7)

Data are presented as mean ± standard deviation (*x̄*±s) for continuous variables and *n* (%) for categorical variables, “—” indicates no corresponding data in the group.

### 3.2 Interobserver agreement

The interobserver agreement for image quality scores was excellent for PETRA-MRA (*κ* = 0.82) and good for TOF-MRA (*κ* = 0.71), indicating reliable scoring consistency between readers.

### 3.3 Image quality of the parent artery

Across both treatment groups, PETRA-MRA consistently demonstrated significantly higher image quality scores compared with TOF-MRA at both T0 and T1 time points (all P_FDR < 0.01). Overall image quality in the flow-diverter group was superior to that in the SAC group (P_FDR < 0.001) (Table 2).

**Table 2:** Parent artery image quality scores of TOF-MRA and PETRA-MRA.

Interventional treatment method	Imaging modality	Time point	Parent artery image quality score [Median (interquartile range), M(IQR)]	Adjusted *p* (FDR)^b^
Flow diverter stent group	TOF-MRA	T0	1.5 [1.0]	Pb = 0.009; Pa1 = 0.443
		T1	3.0 [2.25]	Pc = 0.008; Pa3 < 0.001
	PETRA-MRA	T0	3.5 [1.25]	Pb = 0.009; Pa2 = 0.956
		T1	5.0 [1.0]	Pc = 0.008; Pa4 < 0.001
	TOF-MRA (T0 vs. T1)	—	—	Pd = 0.008
	PETRA-MRA (T0 vs. T1)	—	—	Pe = 0.004
Stent-assisted coil embolization group	TOF-MRA	T0	1.0 [1.0]	Pb < 0.001; Pa1 = 0.443
		T1	1.0 [1.0]	Pc < 0.001; Pa3 < 0.001
	PETRA-MRA	T0	3.0 [1.0]	Pb < 0.001; Pa2 = 0.956
		T1	4.0 [1.0]	Pc < 0.001; Pa4 < 0.001
	TOF-MRA (T0 vs. T1)	—	—	Pd = 0.356
	PETRA-MRA (T0 vs. T1)	—	—	Pe = 0.004

*p* values were adjusted using the Benjamini–Hochberg FDR procedure (*q* = 0.05); Pa1: Comparison between the two interventional groups for TOF-MRA at T0, Pa2: Comparison between the two interventional groups for PETRA-MRA at T0, Pa3: Comparison between the two interventional groups for TOF-MRA at T1, Pa4: Comparison between the two interventional groups for PETRA-MRA at T1, Pb: Comparison between TOF-MRA and PETRA-MRA at T0 (within the same interventional group), Pc: Comparison between TOF-MRA and PETRA-MRA at T1 (within the same interventional group); Pd: Comparison between T0 and T1 for TOF-MRA (within the same interventional group); Pe: Comparison between T0 and T1 for PETRA-MRA (within the same interventional group). TOF-MRA, Time-of-Flight Magnetic Resonance Angiography; PETRA-MRA, Pointwise Encoding Time Reduction with Radial Acquisition Magnetic Resonance Angiography; T0, 1 day postoperatively; T1, 6 months postoperatively; FDR, false discovery rate.

In the flow-diverter group, TOF-MRA showed improved image quality at T1 compared with T0; however, no significant difference was observed between T0 and T1 in the SAC group. In contrast, PETRA-MRA demonstrated significantly better image quality at T1 than at T0 in both treatment groups (P_FDR = 0.004).

At T1, both TOF-MRA and PETRA-MRA yielded significantly higher image quality scores in the flow-diverter group relative to the SAC group (P_FDR < 0.001).

**Figure 3:**
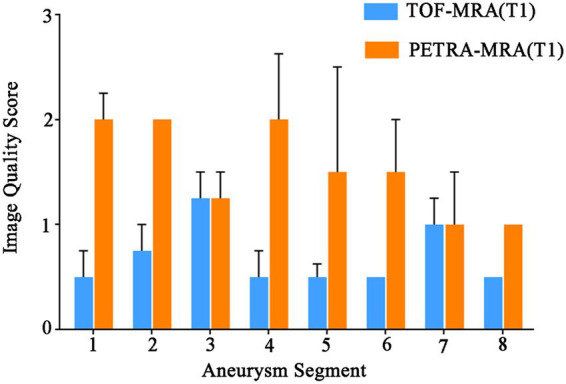
Image quality scores of the treated segment arteries in TOF-MRA and PETRA-MRA at T1. Error bars represent the interquartile range. 1: Ophthalmic segment of the internal carotid artery, 2: Posterior communicating segment of the internal carotid artery, 3: Cavernous segment of the internal carotid artery, 4: Middle cerebral artery, 5: Anterior cerebral artery, 6: Anterior communicating segment, 7: Basilar artery, 8: Posterior cerebral artery. T1, 6 months postoperatively; PETRA-MRA, pointwise encoding time reduction with radial acquisition magnetic resonance angiography; TOF-MRA, time-of-flight magnetic resonance angiography.

PETRA-MRA demonstrated superior image quality across all arterial segments at T1 (Figure 3). Notably, the ophthalmic segment, posterior communicating artery segment, and middle cerebral artery segment achieved image quality comparable to DSA. In the parsimonious ordinal logistic regression model including segment category, stent type, maximum aneurysm diameter, and age, none of the covariates showed a statistically significant independent association with PETRA-MRA image quality at T1 (all *p* > 0.05) (Table 3).

**Table 3:** Parsimonious cumulative logit regression analysis of factors associated with PETRA-MRA image quality at T1.

Variables	Regression coefficient	Standard error (SE)	*p* value	95% Confidence interval (*β*, BCa)
Segment (Ophthalmic vs. Other)	1.294	4.681	0.181	(−16.490 33.183)
Segment category (PCom vs. Other)	0.357	4.556	0.358	(−17.685 32.309)
Stent type (Enterprise vs. non-Enterprise)	−0.057	−0.807	NA	(−17.292 16.850)
Max aneurysm diameter (per mm)	−0.172	−0.645	0.089	(−0.568, −0.091)
Age (per year)	0.021	0.010	0.769	(−0.077, 0.051)

PETRA-MRA image quality scores at T1 were entered as the dependent variable, and a cumulative logit regression model was fitted. A parsimonious multivariable model including segment category, stent type, maximum aneurysm diameter, and age was fitted. Bootstrap resampling with BCa confidence intervals (1,000 iterations) was applied to derive 95% confidence intervals. NA indicates that bootstrap-based OR was not estimable due to sparse category counts. T1, 6 months postoperatively; BCa, bias-corrected and accelerated; Pcom, posterior communicating artery.

### 3.4 Evaluation of aneurysm occlusion status

Aneurysm occlusion was assessed using a binary scale. In the flow-diverter group, complete occlusion increased from 21.4% at T0 to 71.4% at T1. In the SAC group, the complete occlusion rate increased from 80.6% to 91.7%, showing an expected improvement over time (Table 4).

**Table 4:** Evaluation results of aneurysm occlusion status by DSA, TOF-MRA, and PETRA-MRA.

Surgical method	Aneurysm occlusion status	Time point	DSA [*n* (%)]	TOF-MRA [*n* (%)]	PETRA-MRA [*n* (%)]
Flow diverter stent (*n* = 28)	Complete occlusion	T0	6 (21.4)	10 (35.7)	8 (28.6)
		T1	20 (71.4)	22 (78.6)	20 (71.4)
	Incomplete occlusion	T0	22 (78.6)	18 (64.3)	20 (71.4)
		T1	8 (28.6)	6 (21.4)	8 (28.6)
Stent-Assisted Coil Group (*n* = 72)	Complete occlusion	T0	58 (80.6)	64 (88.9)	56 (77.8)
		T1	66 (91.7)	65 (90.3)	65 (90.3)
	Incomplete occlusion	T0	14 (19.4)	8 (11.1)	16 (22.2)
		T1	6 (8.3)	7 (9.7)	7 (9.7)

Data presentation: values are expressed as “*n* (%),” where “*n*” is the number of aneurysms with the corresponding occlusion status, and “%” is the percentage of such aneurysms relative to the total number in the group. DSA, Digital Subtraction Angiography; TOF-MRA, Time-of-Flight Magnetic Resonance Angiography; PETRA-MRA, Pointwise Encoding Time Reduction with Radial Acquisition Magnetic Resonance Angiography; T0, 1 days postoperatively; T1, 6 months postoperatively.

### 3.5 Diagnostic performance of PETRA-MRA versus TOF-MRA

Using DSA as the reference standard, PETRA-MRA demonstrated superior diagnostic performance compared with TOF-MRA at both time points across treatment groups (Table 5).

**Table 5:** Diagnostic efficacy of PETRA-MRA and TOF-MRA for postoperative aneurysm occlusion assessment.

Surgical method	Comparison method	Sensitivity, % (95% CI)	Specificity, % (95% CI)	PPV, % (95% CI)	NPV, % (95% CI)	Accuracy, % (95% CI)
Flow diverter stent group	TOF-MRA (T0)	81.82 (48.23–97.72)	100 (29.24–100)	100 (66.37–100)	60 (14.66–94.73)	85.71 (57.19–98.18)
	PETRA-MRA (T0)	90.91 (58.72–99.47)	100 (29.24–100)	100 (69.15–100)	75 (19.41–99.37)	92.86 (66.14–99.82)
	TOF-MRA (T1)	75 (19.41–99.37)	90 (55.50–99.75)	75 (19.41–99.37)	90 (55.50–99.75)	85.71 (57.19–98.18)
	PETRA-MRA (T1)	100 (51.76–100)	90 (55.50–99.75)	80 (28.36–99.49)	100 (66.37–100)	92.86 (66.14–99.82)
Stent-assisted coil embolization group	TOF-MRA (T0)	35.71 (16.34–61.24)	93.1 (83.63–97.33)	55.56 (26.66–81.12)	85.71 (74.98–92.53)	81.94 (71.38–89.11)
	PETRA-MRA (T0)	92.86 (68.53–98.73)	94.83 (85.87–98.20)	81.25 (57.02–93.41)	98.21 (90.55–93.41)	94.44 (86.38–97.82)
	TOF-MRA (T1)	80 (43.65–96.99)	94.74 (87.52–98.41)	66.67 (30.57–86.32)	97.3 (91.70–99.72)	93.02 (86.38–97.82)
	PETRA-MRA (T1)	100 (61.02–100)	97.37 (91.91–99.73)	83.33 (48.69–97.43)	100 (94.42–100)	97.67 (92.51–99.77)

Diagnostic gold standard: All efficacy indicators are calculated with digital subtraction angiography (DSA) as the reference standard. TOF-MRA, Time-of-Flight Magnetic Resonance Angiography; PETRA-MRA, Pointwise Encoding Time Reduction with Radial Acquisition Magnetic Resonance Angiography; PPV, Positive Predictive Value; NPV, Negative Predictive Value. T0, 1 days postoperatively; T1, 6 months postoperatively; CI, Confidence Interval.

## 4 Discussion

In this prospective cohort study, we systematically compared the performance of PETRA-MRA and TOF-MRA at two key postoperative time points—early (1 days, T0) and mid-term follow-up (6 months, T1)—using DSA as the reference standard. We further examined whether imaging performance differed between treatment modalities, namely flow-diverter monotherapy and SAC. Our findings demonstrate that PETRA-MRA provides consistently superior parent artery visualization and diagnostic sensitivity compared with TOF-MRA, with this advantage becoming particularly pronounced at the mid-term follow-up and in patients treated with FD.

Several UTE – based techniques (18) have been proposed to reduce susceptibility artifacts in postoperative aneurysm imaging. Among these, Silent MRA and PETRA-MRA represent two commonly used vendor-specific implementations. Silent MRA (19), developed by GE Healthcare, typically combines arterial spin labeling (ASL) with UTE (TE = 0.016 ms) acquisition to visualize blood flow with minimal susceptibility artifacts. Silent MRA has been validated in several postoperative aneurysm imaging studies with outstanding performance, offering valuable guidance for clinical diagnosis and management (19, 20). In contrast, PETRA-MRA, implemented on Siemens platforms, is a relatively newer technique that employs a radial acquisition scheme with point-by-point encoding of the central k-space. It enables an ultra-short effective echo time (TE = 0.07 ms) and improves imaging robustness near metallic implants. Although the technical implementations differ, both approaches belong to the broader category of artifact-reduced non-contrast MRA techniques and share the common goal of improving visualization of vessels adjacent to metallic devices. Thus, PETRA-MRA can be regarded as a suitable alternative to Silent MRA in institutions equipped exclusively with Siemens MRI scanners.

In our study, PETRA-MRA consistently demonstrated higher image quality scores than TOF-MRA at both T0 and T1 across treatment modalities, consistent with previous reports (13). The technical basis for this superiority lies in PETRA-MRA’s UTE combined with radial acquisition, which substantially reduces susceptibility artifacts, improves vessel boundary delineation, and enhances visualization near metal implants (21, 22). In contrast, TOF-MRA is inherently susceptible to signal loss due to radiofrequency shielding, saturation effects, and stent-induced magnetic field distortions, which are particularly problematic in SAC (23). Furthermore, no anatomical segment category, stent type, aneurysm size, or age demonstrated a stable independent association with PETRA-MRA image quality at T1, suggesting that the imaging advantage of PETRA-MRA is broadly consistent across segments and device types (12). Both PETRA-MRA and TOF-MRA demonstrated time-dependent improvement in image quality, with T1 assessments outperforming T0. Several mechanisms likely contribute to this trend: endothelialization around the stent is more complete at T1, reducing local flow turbulence (24); hemodynamic stability increases over time, diminishing thrombus-related signal inconsistencies; and in flow-diverter–treated patients, progressive vessel remodeling leads to smoother luminal contours, further enhancing visualization. Collectively, these factors allow PETRA-MRA to approximate DSA more closely during mid-term follow-up, especially in flow-diverter cases.

Our findings also corroborate existing evidence that aneurysm occlusion follows a time-dependent trajectory. Progressive endothelialization and sustained low-shear, low-flow conditions promote ongoing thrombosis at the aneurysm neck (25) reported gradual improvement in occlusion rates over time, and a meta-analysis (26) by noted complete occlusion rates as high as 96% at 5 years. Consistent with these observations, the complete occlusion rate in our study increased from 21.4% to 71.4% in the flow-diverter group and from 80.6% to 91.7% in the SAC group between T0 and T1. These findings reinforce the temporal nature of aneurysm healing and emphasize the need for structured follow-up imaging, particularly given that healing dynamics may differ between treatment modalities.

When assessing aneurysm occlusion with DSA as the reference standard, PETRA-MRA demonstrated markedly better diagnostic performance than TOF-MRA. PETRA-MRA achieved higher sensitivity and overall accuracy at both time points, with sensitivity reaching 100% at T1 in both treatment groups. In the SAC group at T0, TOF-MRA showed notably low sensitivity (35.71%), which may lead to an increased risk of missed residual aneurysms. In contrast, PETRA-MRA maintained consistently high sensitivity (92.86% at T0 and 100% at T1) and high accuracy (94.44% at T0 and 97.67% at T1), with values much closer to those of DSA. Although TOF-MRA retained relatively high specificity in most subgroups, its lower sensitivity limited its reliability for detecting residual aneurysm filling. In the FD group, however, the accuracy of TOF-MRA and PETRA-MRA was similar at both time points, which may be related to the relatively small sample size in our study. This limitation likely arises from the inability of TOF-MRA to accurately detect small remnants in the presence of metallic susceptibility artifacts (27).

From a clinical perspective, PETRA-MRA is valuable for postoperative surveillance following endovascular treatment of intracranial aneurysms. It has a scan time comparable to TOF-MRA and exhibits excellent compatibility and stability in patients with implanted devices. Although it has limitations such as subtraction errors due to head position mismatch and slightly degraded imaging under low-flow conditions, PETRA-MRA enables rapid assessment of stent position and embolization status in the early postoperative period, facilitating the detection of early complications. For mid- and long-term follow-up, it reduces susceptibility artifacts and clearly delineates the parent artery, making it a reliable noninvasive imaging modality. Therefore, PETRA-MRA can serve as a first-line screening tool in routine follow-up, with DSA reserved for inconclusive cases or prior to retreatment. This strategy establishes an efficient noninvasive screening → invasive confirmation workflow, minimizing repeated invasive angiography while maintaining diagnostic accuracy.

However, several limitations of the present study should be acknowledged. First, it was conducted at a single center with a moderate sample size (100 patients included in the final analysis), which may introduce selection bias. Second, we did not perform subgroup analyses by stent type (e.g., Enterprise, Neuroform Atlas) due to insufficient case numbers, preventing a more granular evaluation of device-specific imaging performance. Third, baseline characteristics differed between treatment groups, reflecting the real-world clinical indications for FD placement versus SAC. These differences may introduce potential confounding when comparing imaging performance across treatment modalities. Lastly, the follow-up period was limited to 6 months; longer-term performance and stability of PETRA-MRA remain to be validated. Future work will involve multicenter studies with larger cohorts, stratified analyses by stent design, aneurysm morphology, and vessel characteristics, as well as extended follow-up (≥12 months) to assess long-term diagnostic durability. Further, integrating PETRA-MRA with artificial intelligence–based quantitative residual aneurysm analysis may provide automated, high-precision assessment and further enhance follow-up efficiency.

## 5 Conclusion

This prospective study demonstrates that PETRA-MRA offers favorable advantages over TOF-MRA in the postoperative follow-up of intracranial aneurysms, including improved parent artery image quality, higher sensitivity for residual aneurysm detection, and better overall diagnostic accuracy. Although TOF-MRA shows relatively high accuracy and specificity in the FD group at the early time point, PETRA-MRA provides more stable and reliable performance, particularly under metallic susceptibility artifacts. These findings support PETRA-MRA as a reliable and effective imaging modality—particularly for mid-term and longer-term follow-up—and suggest that it may serve as the preferred noninvasive alternative to DSA in the surveillance of intracranial aneurysm treatment outcomes.

## Data Availability

The raw data supporting the conclusions of this article will be made available by the authors, without undue reservation.
